# Quercetin Attenuates Chronic Ethanol-Induced Hepatic Mitochondrial Damage through Enhanced Mitophagy

**DOI:** 10.3390/nu8010027

**Published:** 2016-01-05

**Authors:** Xiao Yu, Yanyan Xu, Shanshan Zhang, Jian Sun, Peiyi Liu, Lin Xiao, Yuhan Tang, Liegang Liu, Ping Yao

**Affiliations:** 1 Department of Nutrition and Food Hygiene, School of Public Health, Tongji Medical College, Huazhong University of Science and Technology, Wuhan 430030, China; yuxiao775@126.com (X.Y.); yyx@hust.edu.cn (Y.X.); 13035130764@163.com (S.Z.); ycsjian@163.com (J.S.); liupeiyi85@126.com (P.L.); xiaolin0210@hust.edu.cn (L.X.); lgliu@mails.tjmu.edu.cn (L.L.); 2 Hubei Key Laboratory of Food Nutrition and Safety, School of Public Health, Tongji Medical College, Huazhong University of Science and Technology, Wuhan 430030, China; 3 Ministry of Education Key Laboratory of Environment, School of Public Health, Tongji Medical College, Huazhong University of Science and Technology, Wuhan 430030, China

**Keywords:** mitochondrial damage, mitophagy, quercetin, FoxO3a, AMPK, ERK2

## Abstract

Emerging evidence suggested mitophagy activation mitigates ethanol-induced liver injury. However, the effect of ethanol on mitophagy is inconsistent. Importantly, the understanding of mitophagy status after chronic ethanol consumption is limited. This study evaluated the effect of quercetin, a naturally-occurring flavonoid, on chronic ethanol-induced mitochondrial damage focused on mitophagy. An ethanol regime to mice for 15 weeks (accounting for 30% of total calories) led to significant mitochondrial damage as evidenced by changes of the mitochondrial ultrastructure, loss of mitochondrial membrane potential and remodeling of membrane lipid composition, which was greatly attenuated by quercetin (100 mg/kg.bw). Moreover, quercetin blocked chronic ethanol-induced mitophagy suppression as denoted by mitophagosomes-lysosome fusion and mitophagy-related regulator elements, including LC3II, Parkin, p62 and voltage-dependent anion channel 1 (VDAC1), paralleling with increased FoxO3a nuclear translocation. AMP-activated protein kinase (AMPK) and extracellular signal regulated kinase 2 (ERK2), instead of AKT and Sirtuin 1, were involved in quercetin-mediated mitophagy activation. Quercetin alleviated ethanol-elicited mitochondrial damage through enhancing mitophagy, highlighting a promising preventive strategy for alcoholic liver disease.

## 1. Introduction

Alcohol abuse has been a leading risk factor for morbidity, disability and mortality worldwide. Approximately 5.9% of global deaths and 5.1% of global disability-adjusted life-years were attributable to excessive alcohol consumption [1]. As an organ principally responsible for alcohol metabolism, the liver is particularly susceptible to alcohol toxicity. Alcoholic liver disease (ALD) is an increasing global public health issue. ALD usually begins with nearly asymptomatic steatosis, but can stealthily progress to steatohepatitis, fibrosis and even to almost irreversible cirrhosis and hepatocellular carcinoma (HCC) [2]. ALD is one of the most common leading causes of HCC and a major indication for liver transplantation worldwide [3]. Therefore, it is imperative to explore the underlying mechanism for the onset and progression of ALD and specific early intervention strategies.

Mitochondrial damage is the early pathological characteristics of ALD [4], but accompanies the various stages of ALD [5,6,7,8]. Mitophagy suppression increased mitochondrial damage and further aggravated ethanol-induced liver disease, while enhancing mitophagy potentially protected against ethanol-imposed hepatotoxicity [5,9]. However, limited mitophagy activation failed to offer its hepatic protection in rats following five days of ethanol intake at a dose of 5 g/kg.bw [10]. Thus, loss of mitophagy capacity or limited induction of mitophagy might be the underlying initiating factor for ALD progression. Increasing evidence proved that mitophagy activation reduced acute and chronic ethanol-induced hepatotoxicity, revealing different levels of mitochondrial dysfunction [5,9,10,11]. Although direct evidence has yet to be sought, the status of mitophagy could be different between acute and chronic ethanol-induced liver damage. Most importantly, it is also possible that mitophagy function eventually deteriorates in a more severe regimen.

Quercetin, a plant-derived flavonoid abundant in various vegetables, fruits, herbs and red wine, exhibited prominent anti-oxidative and anti-inflammatory activities [12,13,14]. Our preliminary study demonstrated that quercetin ameliorated ethanol-induced hepatic mitochondrial damage [15]. Furthermore, quercetin-induced autophagy was proven to attenuate renal ischemia/reperfusion injury and suppress tumor growth *in vitro* and *in vivo* [16,17,18]. These emerging studies revealed a potential protection for quercetin against ethanol-induced mitochondrial damage through activating mitophagy. In addition, evidence for mitophagy induction by resveratrol, a polyphenolic autophagy inducer similar to quercetin, in maintaining myocardial mitochondria homeostasis, further supported this hypothesis [19]. Distinct signaling pathways, including AMP-activated protein kinase (AMPK) and Sirtuin1 (SIRT1) following renal ischemia reperfusion injury [16,19], as well as AKT and extracellular signal-regulated kinase (ERK) in tumor cells [18,20], were implicated in quercetin and other polyphenol-mediated autophagy induction. However, in the context of ethanol-induced liver injury, an integration of oxidative stress, endoplasmic reticulum stress and inflammatory response, it remained elusive whether and how these various signaling pathways were involved in mitophagy regulation by quercetin.

## 2. Materials and Methods

### 2.1. Chemicals

Ethanol and quercetin (≥98%, HPLC) were purchased from Zhenxing Chemical Factory (Shanghai, China) and Sigma-Aldrich, respectively. Anti-GAPDH rabbit polyclonal antibody, anti-FoxO3a rabbit monoclonal antibody, anti-ERK2 rabbit polyclonal antibody, anti-PI3K rabbit monoclonal antibody, anti-AKT rabbit monoclonal antibody, anti-p-AKT rabbit monoclonal antibody, anti-AMPK rabbit monoclonal antibody, anti-p62 rabbit monoclonal antibody, anti-Parkin mouse monoclonal antibody, anti-rabbit IgG (secondary antibody) and anti-mouse IgG (secondary antibody) were obtained from Cell Signaling Technology. Anti-SIRT1 and anti-VDAC1 mouse monoclonal antibodies were provided by abcam. Anti-LC3II rabbit polyclonal antibody was purchased from Sigma-Aldrich. Western blotting detecting reagents (ECL) and reblot buffer were provided by Chemicon (Temecula, CA, USA). Acetyl chloride (purity > 98%) and methanol (HPLC grade) were purchased from Sigma-Aldrich. Chloroform and n-hexane (HPLC grade) were provided by Fisher Scientific, Leicestershire, UK. Other chemicals and organic solvents were of analytical grade and purchased from a local reagent retailer.

### 2.2. Animal Treatment

Male C57BL/6J mice weighing 18–20 g were obtained from Sino-British Sippr/BK (Shanghai, China). The animals were housed in stainless steel cages at a controlled room temperature (25 ± 2 °C) and relative humidity (65%–75%) with a 12:12 light dark cycle. The animals were taken care of according to the Guiding Principles in the Care and Use of Laboratory Animals published by the U.S. National Institutes of Health. Animal experiments described in this study were approved by the Tongji Medical College Council on Animal Care Committee. After one week of acclimatization with chow diet, the animals were randomly divided into 4 groups of 15 animals in each and pair-fed with isocaloric Lieber–DeCarli liquid diets (Beijing HFK Biosicence Co., Ltd, Beijing, China) for 15 weeks as the following: (1) normal control group (Ct), receiving regular Lieber–DeCarli liquids diets; (2) ethanol group (Et), fed with ethanol-containing Lieber–DeCarli liquid diets (30% of total calories as ethanol); (3) quercetin and ethanol group (Qu + Et), receiving ethanol-containing Lieber–DeCarli liquid diets (30% of total calories as ethanol) and administrated quercetin by gavage (100 mg/kg body weight); (4) quercetin control group (Qu), receiving regular Lieber–DeCarli liquid diets and administrated quercetin by gavage (100 mg/kg body weight). The ethanol content of the diet gradually increased over a 12-day period (no ethanol for Day 2, one fourth of the given amount for Days 3–5, half of the given amount for Days 6–9, two thirds of the given amount for Days 10–12 and full amount for the rest). The quercetin was dissolved in physiological saline to obtain an oral suspension.

The mice were sacrificed after an overnight fasting. Fresh liver samples were fixed for histopathology determination, and the quick-frozen ones by liquid nitrogen were used for RT-PCR and Western blot analysis.

### 2.3. Transmission Electron Microscopy

Transmission electron microscopy (TEM) was performed on mice liver according to standard protocols. Briefly, fresh liver fragments (1-mm cubes) were collected carefully, rapidly fixed in 2.5% glutaraldehyde (in phosphate buffer, pH 7.4) and then post-fixed in 1% osmium tetraoxide. Subsequently, the samples were dehydrated by a gradient of increasing concentrations of alcohol, and embedded with Epon 812 resin. Ultrathin sections from Ultramicrotome (Leica UCT, Germany) were stained by uranyl acetate and lead citrate and then detected by transmission electron microscope (FEI Tecnai G2 12, The Netherland). Digital images were captured by an integrated CCD camera.

### 2.4. Mitochondrial Membrane Potential

Mitochondrial membrane potential (∆Ψm) was analyzed by using the fluorescent dye 5,5′6,6′-tetrachloro-1,1′,3,3′-tetraethylbenzimidazol-carbocyanine iodide (JC-1) according to the manufacturer's instructions (Beyotime Institute of Biotechnology, China). Primary hepatocytes were isolated from mice by a two-step collagenase perfusion procedure followed by a Percoll gradient centrifuge for purification [21]. The primary hepatocytes adjusted to 2 × 10^6^ mL^−1^ were washed with phosphate buffered saline (PBS: 8.0 g NaCl, 0.2 g KCl, 3.58 g Na_2_HPO_4_·12H_2_O, 0.24 g KH_2_PO_4_, in 1000 mL deionized water, pH 7.4) before being loaded with JC-1 (25 μg/mL) at 37 °C for 20 min in darkness. After incubation, the cells were rinsed three times with JC-1 dyeing buffer. After removing the supernatant, the cells were re-suspended in 1 mL of PBS. The results of the assay were obtained by flow cytometry (Becton, Dickinson and Company, Franklin Lakes, NJ, USA).

### 2.5. Mitochondrial Membrane Lipid Composition

Mitochondria were isolated from the liver with differential centrifugation [22]. The fresh liver tissues were washed in ice-cold PBS to remove the blood and gently disrupted in fresh homogenization medium containing 225 mM mannitol, 75 mM sucrose, 0.5% fat-free bovine serum albumin, 0.5 mM EGTA and 30 mM Tris-HCL (pH 7.4) in a ratio of 4 mL of buffer per gram of liver. The homogenate was centrifuged twice at 750 g for 5 min to remove cellular debris and nuclei. Post-nuclear supernatant was centrifuged at 10,000× *g* for 10 min to pellet crude mitochondria. The crude mitochondrial pellet was resuspended in isolation medium (250 mM mannitol, 5 mM HEPES (pH 7.4) and 0.5 mM EGTA) and layered on top of 8 mL of Percoll medium (225 mM mannitol, 25 mM HEPES (pH 7.4), 1 mM EGTA and 30% Percoll (v/v)) in a 10-mL polycarbonate ultracentrifuge tube and then centrifuged at 95,000× *g* for 30 min. A dense band containing purified mitochondria was recovered from approximately at the bottom of the tube and washed twice by centrifugation at 6300× *g* for 10 min to remove the Percoll, after which the mitochondria were resuspended in isolation medium for further analysis.

Mitochondrial membrane lipids were extracted using chloroform-methanol (2:1 V/V) [23]. The transmethylation of total fatty acids was performed at 90 °C for 30 min using freshly-prepared 5% acetyl chloride in methanol [24]. The fatty acid methyl esters were separated by Agilent 6890 GC using an HP-INNOWax fused silica capillary column (30 × 0.32, 0.25 μm) and a flame ionization detector; the oven temperature began at 175 °C, was held for 10 min and then increased to 250 °C at 1 °C/min. The injector temperature and detector temperature were set at 250 °C; helium was used as a carrier gas (7.5 mL/min). The injection volume was 1 μL, and the split ratio was 5:1. The fatty acid methyl esters were identified by comparing with authentic standards (NU-CHEK-PREP) and calculated as the percent area of total fatty acids.

### 2.6. Western Blot Analysis

Mice liver tissues were lysed in radio immunoprecipitation assay (RIPA) lysis buffer (1% Triton X-100, 1% deoxycholate, 0.1% sodium dodecyl sulfate (SDS)). Equal amounts of protein extracts were mixed (3:1) with loading buffer for electrophoresis in 10%–12% acrylamide SDS gels and subsequently electroblotted to a polyvinylidene fluoride membrane (Millipore, Billerica, MA, USA) by electrophoresis (Bio-Rad, Hercules, CA, USA). Levels of target proteins were probed with the specific primary antibodies and then incubated with the species-specific second antibodies. The chemiluminescence intensity of the membrane was subsequently detected by the ECL Plus kit in the Western Blotting Detection System (Amersham Biosciences, Little Chalford, UK), and the optical densities of bands were quantified by Gel Pro 3.0 software (Biometra, Goettingen, Germany). Data were corrected to eliminate background noise and standardized to Glyceraldehyde-3-phosphate dehydrogenase (GAPDH) as optical density (OD/mm^2^).

### 2.7. Real-Time Quantitative Polymerase Chain Reaction Analysis

Total RNA was extracted from mice liver tissues using the TRIzol reagent (Ambion^®^, Life Technologies, Austin, TX, USA) according to the manufacturer’s instructions. Messenger RNA (mRNA) expressions of the target genes were quantified by quantitative reverse transcriptase (qRT)-PCR using the SYBR green-based kit (TaKaRa BIO Inc., Dalian, China) with specific primers using an RT-PCR machine (7900HT; Applied Biosystems, Forster, CA, USA). The mRNA level of GAPDH was quantified as an endogenous control, and he results were calculated by a comparative 2^−ΔΔCt^ method. Primer sequences were as follows: USP30 (NM_001033202), 5′-GCTCGCTTCCTGTTAGTAGTTG-3′ and 5′-ACCGTAGGCTGACTGAGAAAG-3′; Sqstm1 (NM_011018), 5′-GCACAGGCACAGAAGACAAG-3′ and 5′-CCACCGACTCCAAGGCTATC-3′; Parkin (NM_016694), 5′-ACCATCAAGAAGACCACCAAG-3′ and 5′-GTTCCACTCACAGCCACAG-3′; FoxO3a (NM_019740.2), 5′-AGCCGTGTACTGTGGAGCTT-3′ and 5′-TCTTGGCGGTATATGGGAAG-3′.

### 2.8. Statistical Analysis

Data were presented as the mean ± SD. The statistical significance of the differences between groups was determined by one-way ANOVA followed by Tukey’s test using SPSS12.0 statistical software (SPSS, Chicago, IL, USA). Differences among groups were considered significant at *p* < 0.05.

## 3. Results

### 3.1. Quercetin Alleviated Chronic Ethanol-Induced Mitochondrial Damage in Mice Liver

**Figure 1:**
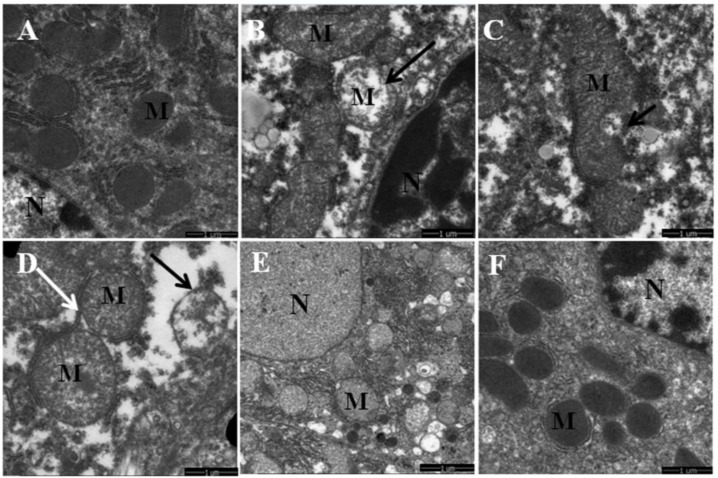
The effects of quercetin on the mitochondrial ultrastructural changes in livers of mice fed with ethanol. (**A**,**F**) normal and quercetin control groups (Ct, Qu); (**B**–**D**) ethanol group (Et); (**E**) quercetin and ethanol groups (Qu + Et); *M*, mitochondria; *N*, nucleus of hepatocyte.

Chronic ethanol treatment caused significant changes in mitochondrial morphology observed by TEM. As shown in Figure 1A,F, mice in normal and quercetin control groups displayed well-developed mitochondria with integral membrane and cristae. However, ethanol treatment induced various degenerative changes in mitochondrial morphology, including abnormal shape, swelling, lacking cristae and destructing inner membranes. Moreover, the other pathologic changes accompanied by mitochondrial damage, including the fractured endoplasmic reticulum and the existence of lipid droplets near the mitochondria, were observed in hepatocytes after ethanol exposure (Figure 1B–D). In contrast, quercetin administration evidently alleviated the morphological abnormalities of mitochondria as a consequence of chronic ethanol treatment (Figure 1E).

**Figure 2:**
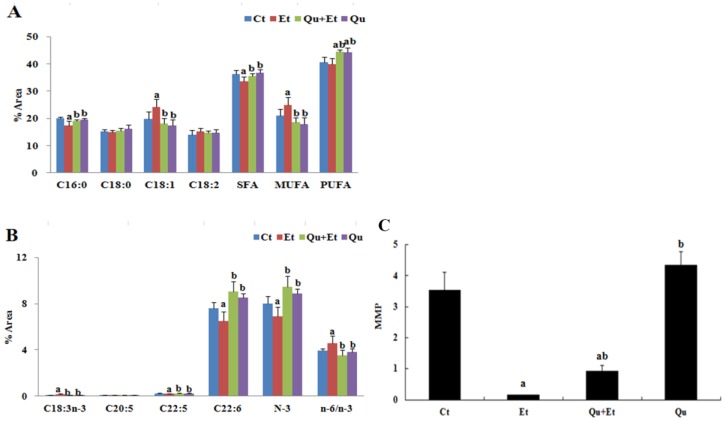
Effects of quercetin on the mitochondrial membrane lipid composition and membrane potential in livers of mice fed with ethanol. (**A**) The levels of C16:0, C18:0, C18:1, C18:2, SFA (given by C12:0 + C14:0 + C16:0 + C18:0 + C20:0 + C22:0 + C24:0), MUFA (given by C14:1 + C16:1 + C18:1 + C20:1 + C22:1 + C24:1) and PUFA (given by C18:2 + C18:3*n*-6 + C18:3*n*-3 + C20:2 + C20:3 + C20:4 + C20:5 + C22:4 + C22:5 + C22:6); (**B**) the levels of C18:3, C20:5, C22:5, C22:6, *n*-3PUFA (given by C18:3 + C20:5 + C22:5 + C22:6) and the ratio of *n*-6/*n*-3 (given by C18:2*n*-6 + C20:4*n*-6 + C22:4*n*-6 to C18:3*n*-3 + C20:5*n*-3 + C22:5*n*-3 + C22:6*n*-3); (**C**) the mitochondrial membrane potential (MMP). The levels of fatty acids were expressed as the percent area of total fatty acids. Results are expressed as the mean ± SD. A significant difference (*p* < 0.05) is identified by different letters: a, *vs.* the control group (Ct); b, *vs.* the ethanol group (Et).

The remodeling of mitochondrial membrane lipids may affect the function of specific membrane proteins and, consequently, lead to mitochondrial dysfunction. As depicted in Figure 2A,B, the mitochondrial membrane lipids in the normal mice liver consisted of significantly higher levels of saturated fatty acids (SFAs) and polyunsaturated fatty acids (PUFAs), whereas the contents of monounsaturated fatty acids (MUFAs) were much lower than those of corresponding dietary lipids, indicating that *de novo* synthesized SFAs are preferred over diet-derived MUFAs as the substrate for the synthesis of mitochondrial membrane phospholipids. Nevertheless, chronic ethanol exposure induced marked changes in particular fatty acids. Oleic acid (C18:1, *n*-9), a bonafide MUFA of *de novo* lipogenesis in liver, was significantly rich in mitochondria membrane of ethanol-dosed mice liver in comparison with the normal control (22.36% *versus* 18.25%). Furthermore, an increased mitochondrial *n*-6 to *n*-3 ratio (+16%, *p* < 0.05) and reduced *n*-3 PUFA, especially docosapentaenoic (22:5, *n*-3) and docosahexaenoic (22:6, *n*-6), levels (−21%, *p* < 0.05; −15%, *p* < 0.05) were observed in mice liver following ethanol treatment. Interestingly, we observed a robust effect of ethanol feeding on α-linolenic acid (18:3, *n*-3) accumulation in mitochondria (+112%, *p* < 0.05) and no significant influence on linoleic acid (18:2, *n*-6). These effects were normalized by quercetin administration, with decreased contents of C18:1 (−25%, *p* < 0.05), increased synthesis of C22:5 and C22:6 from C18:3 (+21%, *p* < 0.05; +40%, *p* < 0.05) and a balanced *n*-6 to *n*-3 ratio (3.55 *versus* 4.56) in mitochondrial membrane phospholipids.

Mitochondrial membrane potential was subsequently determined by JC-1, a fluorescent lipophilic cationic probe, to evaluate mitochondrial polarization following chronic ethanol exposure or quercetin intervention. As shown in Figure 2C, chronic ethanol-dosed mice revealed a substantial depolarization of ∆Ψm when compared to the control group (0.2 *versus* 3.4, *p* < 0.05), manifesting a remarkable increase in the percentage of green-fluorescent-positive cells as a result of JC-1 monomers’ accumulation in depolarized mitochondria. However, the loss of ∆Ψm was partially reversed (drastic increase in JC-1 aggregation) when co-treated with quercetin for ethanol-fed mice (0.9 *versus* 0.2, *p* < 0.05), although both values were still lower than the normal and quercetin control groups.

### 3.2. Quercetin Enhanced Chronic Ethanol-Mediated Mitophagy Suppression in Mice Liver

**Figure 3:**
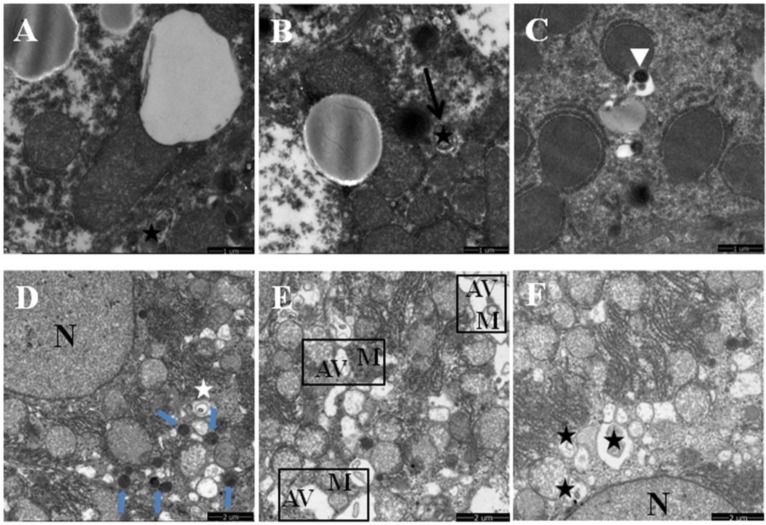
Effects of quercetin on the mitophagy by transmission electron microscopy in livers of mice fed with ethanol. (**A**–**C**) Ethanol group (Et); (**D**–**F**) quercetin and ethanol groups (Qu + Et); the black star in (**A**,**B**,**E**,**F**) demonstrates the early autophagic vacuoles (mitophagosomes); the white triangle in (**C**) shows the late autophagic vacuoles (autolysosomes); the short blue arrow in (**D**) marks lysosomes; *M*, mitochondria; *N*, nucleus of hepatocyte.

Mitophagy is the selective degradation of mitochondria by autophagy to promote mitochondrial turnover and prevent accumulation of dysfunctional mitochondria, which can lead to cellular degeneration. As anticipated, chronic ethanol exposure inhibited the autophagic elimination of damaged mitochondria. The total number of early autophagic vacuoles (autophagosomes or mitophagosomes) and autolysosomes characterized by degenerated mitochondria and lysosomes was decreased in ethanol-dosed mice liver. Accordingly, such accumulated mitochondria were partly or totally enclosed by fractured endoplasmic reticulum-like membranes and became fused, self-dissolved (Figure 1B–D, or occasionally sequestrated inside autophagic vacuoles (Figure 3A–C). On the contrary, the mitophagosomes, autolysosomes and lysosomes were frequently observed in liver of mice co-treated with quercetin and ethanol (Figure 3D–F), indicating an enhancing mitochondrial degradation by autophagy activated by quercetin.

**Figure 4:**
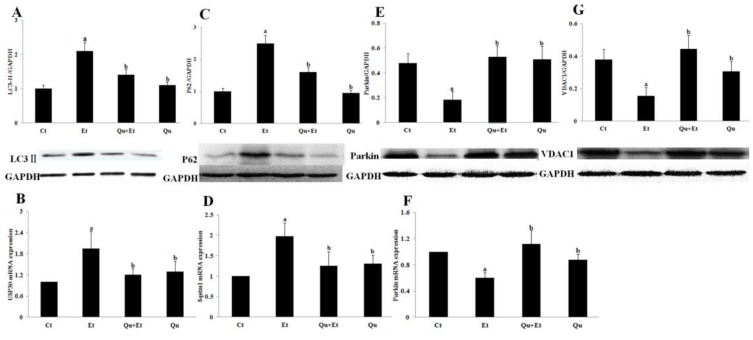
Effects of quercetin on the expression of mitophagy-related regulator elements in livers of mice fed with ethanol. Messenger RNA expressions of USP30 (**B**), Sqstm1 (**D**) and Parkin (**F**) were determined with qRT-PCR. Protein levels of LC3II (**A**), P62 (**C**), Parkin (**E**) and VDAC1 (**G**) were analyzed by Western blotting. Results are expressed as the mean ± SD (six independent experiments for qRT-PCR, three for Western blotting). A significant difference (*p* < 0.05) is identified by different letters: a, *vs.* the control group (Ct); b, *vs.* the ethanol group (Et).

**Figure 5:**
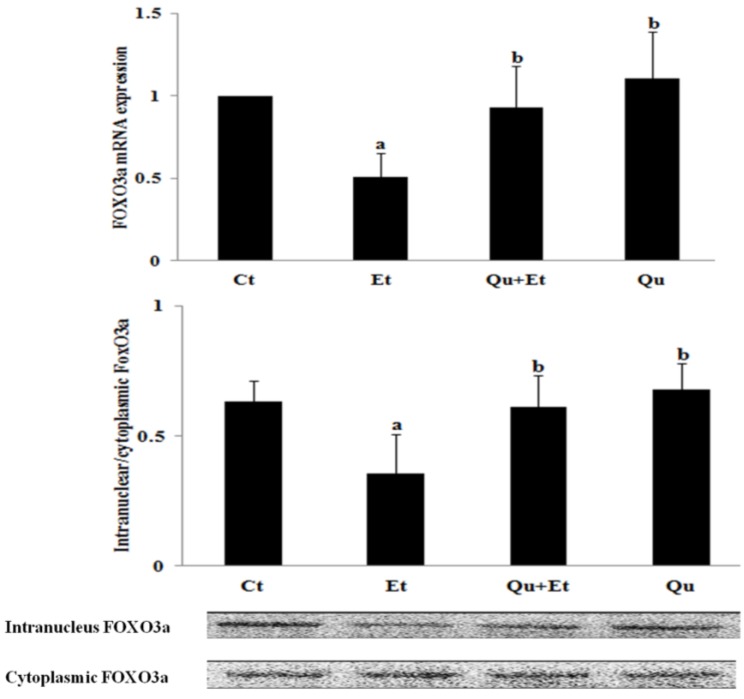
Effects of quercetin on the expression of FoxO3a in livers of mice fed with ethanol. A significant difference (*p* < 0.05) is identified by different letters. Hepatic mRNA expression of FoxO3a was measured by qRT-PCR (**A**); Western blotting was used to determine protein levels of intranuclear and cytoplasmic FoxO3a (**B**). Results are expressed as the mean ± SD (six independent experiments for qRT-PCR, three for Western blotting). a, *vs.* the control group (Ct); b, *vs.* the ethanol group (Et).

Ethanol-mediated mitophagy suppression in mice liver was further confirmed by the changes of mitophagy-related regulator elements. As shown in Figure 4A, Western blot analysis revealed a significant increase in LC3II expression induced by chronic ethanol exposure when compared to the control group. Additionally, the mRNA and protein expressions of p62, an autophagic substrate protein that was usually degraded by autophagy, were remarkably increased after chronic ethanol treatment (Figure 4C,D). However, the upregulation of LC3II and p62 was blocked when mice were co-treated with ethanol and quercetin. Importantly, our data showed that a chronic ethanol regime inhibited the expression of Parkin, an E3 ubiquitin ligase required for mitophagy when translocated from the cytosol to damaged mitochondria (Figure 4E,F). There was a significant decrease in the expression of VDAC1, the mitochondrial outer membrane protein required for Parkin-dependent mitophagy, in liver of mice treated with ethanol (Figure 5G). Furthermore, the mRNA expression of mitochondrial deubiquitinase ubiquitin-specific protease 30 (Usp30), which antagonized Parkin-mediated mitophagy, was significantly upregulated in liver of mice after chronic ethanol exposure (Figure 4B). Quercetin administration markedly attenuated the unfavorable changes of Parkin, Usp30 and VDAC1 induced by ethanol. These results suggested that quercetin directed a protective effect on chronic ethanol-induced mitochondrial damage in liver of mice through enhancing Parkin-dependent mitophagy.

### 3.3. Increased Nuclear Translocation of FoxO3a Potentially Mediated the Protective Effect of Quercetin against Mitophagy Suppression

FoxO3a is a key factor in regulating ethanol-induced autophagy and cell survival. Therefore, we examined whether the changes in FoxO3a transcriptional activity were involved in chronic ethanol-induced mitochondrial damage and mitophagy suppression. As manifested in Figure 5, chronic ethanol exposure dramatically suppressed the mRNA expression, as well as nuclear retention of FoxO3a. Nevertheless, quercetin administration significantly increased FoxO3a expression and nuclear translocation. Collectively, these data indicated that quercetin-mediated enhancement of FoxO3a transcriptional activity was involved in the protective effect against mitophagy suppression induced by ethanol.

### 3.4. AMPK and ERK2, Instead of PI3K/AKT and SIRT1, Were Involved in Quercetin-Mediated Mitophagy Activation

**Figure 6:**
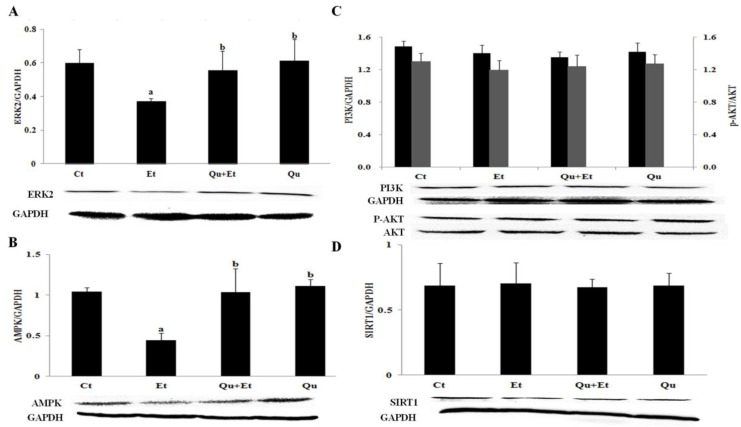
Effects of quercetin on the expression of ERK2 (**A**), AMPK (**B**), PI3K, p-AKT/AKT (**C**) and SIRT1 (**D**) in livers of mice fed with ethanol. Results are expressed as the mean ± SD (three independent experiments for Western blotting). A significant difference (*p* < 0.05) is identified by different letters: a, *vs.* the control group (Ct); b, *vs.* the ethanol group (Et).

To further explore the potential molecular mechanism involved in mitophagy induction by quercetin, the protein expressions of AMPK, PI3K/AKT, ERK2 and SIRT1 were determined in mice liver. The results showed that the expressions of AMPK and ERK2 significantly decreased after chronic ethanol exposure, while PI3K/AKT and SIRT1 protein levels remained unchanged in comparison with the control group (Figure 6). Importantly, quercetin supplementation partially reversed the effect of ethanol regime on AMPK and ERK2, yet had no effect on PI3K/AKT and SIRT1. Altogether, quercetin treatment attenuated ethanol-induced mitophagy suppression partially dependent on AMPK and ERK2.

## 4. Discussion

Mitochondria contribute to ethanol metabolism via acetaldehyde oxidation and nicotinamide adenine dinucleotide (NAD^+^) replenishment by NADH oxidation in the respiratory chain. However, ethanol administration causes mitochondrial DNA damage and excessive production of reactive oxygen species (ROS), negatively impacting mitochondrial biogenesis and function. Therefore, mitochondrial dysfunction predominates in ethanol-induced liver injury [25]. In the current study, chronic ethanol overload caused various degenerative changes of liver histology, particularly on mitochondria with abnormal shape, swelling, lacking cristae, destructing inner membranes and autolysis. Additionally, many lipid droplets and fractured endoplasmic reticulum-like membranes were near the mitochondria. Furthermore, substantial collapse of mitochondrial membrane potential in response to ethanol stress was observed in mice liver. Our findings were partly consistent with earlier studies [5,26]. Most importantly, we further found that ethanol-induced mitochondrial damage and dysfunction were intimately related to the remodeling of mitochondrial membrane phospholipids with an enhanced amount of C18:1 and decreased content of C16:0. Previous studies reported that increased contents of C16:0, C18:0 and C18:1 were observed in mitochondrial phospholipids from rats following five weeks of ethanol treatment [27]. This apparent discrepancy suggested that extending ethanol exposure time could further upregulate *de novo* lipogenesis and subsequent acyl chain elongation and desaturation within the mice liver. Notably, our data revealed for the first time that ethanol led to increased levels of C18:2 and C18:3 and reduced concentrations of C22:5 and C22:6 in mitochondrial phospholipids, due to inhibited Δ-5 or Δ-6 desaturase activities in liver after ethanol treatment [28]. The altered fatty acid composition in mitochondria was expected to influence lipid re-distribution within membrane, especially the cardiolipin (CL) considered as an important contributing factor in mitochondrial dysfunction in several physiopathological situations [29]. Quercetin supplementation greatly reversed ethanol-induced mitochondrial damage evidenced by decreased accumulation of damaged mitochondria and improved mitochondrial morphology and membrane properties. Mechanically, reduced *de novo* fatty acid synthesis and upregulated desaturase activity by quercetin partly explained the favorable effect on mitochondrial membrane lipid composition [30,31,32]. DHA, as a primary fatty acid in mitochondrial phospholipids, was required for the organization and function of membrane proteins [33]. Therefore, the increased accumulation of DHA within mitochondrial membrane mediated by quercetin may affect the function of mitochondrial outer membrane protein and benefit the removal of damaged mitochondria. It has also been shown that DHA could increase the capacity for mitochondrial ROS emission without increasing the status of oxidative damage [34]. In addition, the preferential accumulation of quercetin in mice liver, especially in mitochondria, further indicated the potential for alleviating ethanol-induced mitochondrial damage due to the presence of antioxidant pharmacophores such as the OH group at position 3 of the AC-ring, which had the optimal configuration for mitochondrial ROS scavenging [35,36,37].

Besides the protection against mitochondrial damage, it is more important to trigger mitophagy to eliminate in a timely manner the damaged mitochondria [38]. Many studies reported that both acute (4.5–5.0 g/kg.bw for 1–5 days) and chronic (36% of total calories for 10 weeks) ethanol administration stimulated mitophagy, a protective response in mitigating liver injury [5,10,11]. Our study provided direct evidence supporting suppressed mitophagy in the chronic ethanol feeding model. Firstly, ethanol induced mitophagosomes formation and activated the autophagy, but not the autolysosomes characterized by degenerated mitochondria and lysosomes. Furthermore, loss of mitophagy function was confirmed by changes of mitophagy-related regulator elements. The upregulation of LC3II and p62/Sqstm1 further suggested that ethanol stimulated autophagy, but inhibited autophagosome-lysosome fusion due to the reduction in lysosomal function, leading to suppressed mitophagic flux. The expression of unique mitochondrial deubiquitylase (Usp30) was upregulated after chronic ethanol challenge, which was shown to antagonize mitophagy driven by the ubiquitin ligase [39]. VDAC1, the most abundant mitochondrial outer membrane protein, was evidenced as the major mitochondrial substrate of Parkin-dependent mitophagy in neurodegenerative disorder [40,41]. No significant change in VDAC1 expression was found in Parkin knockout mice after chronic plus acute ethanol treatment (36% of total calories for 10 days + 5.0 g/kg.bw), accompanying suppressed mitophagy [9]. In contrast, our data demonstrated that a chronic ethanol regime evidently inhibited the expression of VDAC1 linked with mitophagy inactivation. These discrepant inclusions can be interpreted by more severe mitochondrial damage observed in our study contributing to the degradation of VDAC1. Firstly, chronic ethanol feeding disrupted mitochondrial biogenesis, which might not offset the autophagic degradation of mitochondrial proteins. Alternatively, VDAC1, as a membrane channel, might be susceptibly influenced by the changes of acyl chain length and saturation in mitochondria membrane induced by ethanol exposure [42]. Collectively, the status of mitophagy may alter from acute to chronic ethanol exposure. Mitochondrial ROS, generated during ethanol metabolism, particularly in the liver, was proven to be a significant second messenger involved in the acute ethanol-activated signaling pathway between fragmented mitochondria and autophagy [43]. Mitophagy activation in condition of acute ethanol treatment could lead to a positive increase in mitophagy flux or lysosomal degradation as a compensatory response of hepatocyte injury. However, excessive mitochondrial ROS exposure induced by chronic ethanol feeding may induce negatively-activated mitophagy or blockage of mitophagy due to increased lysosomal damage and ubiquitinated protein aggregates depending on the experimental model, the dose and the experimental condition. Quercetin blocked ethanol-mediated mitophagy suppression by inducing lysosomes biogenesis and accelerating autophagosome-lysosome fusion. Similarly, promoted myocardial mitophagy by resveratrol was demonstrated to prevent heart injuries in rat following ischemic reperfusion [19]. Therefore, quercetin exerted hepatic protection by effectively restoring mitophagy and reopening mitophagy flux.

Many mitophagy effectors, including the mitophagy receptors Nix, Bnip3 and Fundc1, have been identified to participate in the selective clearance of mitochondria [44]. However, Parkin-dependent mitophagy was demonstrated to be a critical mediator of protection against chronic ethanol-induced liver injury [9]. Our data showed that chronic ethanol loading decreased the expression of Parkin corresponding with mitophagy suppression, which was reversed by quercetin administration. FoxO3a, a member of the Forkhead box, subgroup O (FoxO) transcription factors, led to the activation of the Pink/Parkin pathway, potentiating resveratrol-mediated mitophagy in rat following myocardial ischemia reperfusion [19]. FoxO3a nuclear translocation was proven to prevent acute ethanol-induced liver injury through promoting autophagy [45]. Our test revealed that ethanol inhibited FoxO3a expression and nuclear retention, while quercetin evidently upregulated FoxO3a transcriptional activity, leading to enhanced mitophagy. Different signaling pathways, such as AKT [46], SIRT1 [45] and AMPK [47], were proposed to regulate FoxO3a activity and subsequently autophagy induction in acute ethanol-induced hepatotoxicity *in vivo* and *in vitro*, respectively. In addition, ERK-mediated phosphorylation was also involved in impaired FOXO3a expression and activity, contributing to the promotion of tumorigenesis [48]. Our findings found that chronic ethanol exposure reduced the expressions of AMPK and ERK2, instead of PI3K/AKT and SIRT1. It is clear that chronic ethanol-induced liver injury is a multifactorial condition interconnected by varying degrees of oxidative stress, inflammation and abnormalities in lipid metabolism. In this perspective, the regulation of FoxO3a activity is likely more dependent on AMPK and ERK2 under such complex microenvironments induced by a chronic ethanol regime.

## 5. Conclusions

In conclusion, quercetin alleviated chronic ethanol-induced hepatic mitochondrial damage by activated mitophagy, accompanying the increase of FoxO3a nuclear translocation and subsequent upregulation of AMPK and ERK2 expressions. These findings led us to postulate that mitophagy modulation via quercetin may offer a new strategy for prophylactically treating ALD, especially for ethanol-induced hepatic mitochondria damage.
